# The Frequencies of IFNγ+IL2+TNFα+ PPD-Specific CD4+CD45RO+ T-Cells Correlate with the Magnitude of the QuantiFERON® Gold In-Tube Response in a Prospective Study of Healthy Indian Adolescents

**DOI:** 10.1371/journal.pone.0101224

**Published:** 2014-07-03

**Authors:** Synne Jenum, Harleen M. S. Grewal, David A. Hokey, John Kenneth, Mario Vaz, Timothy Mark Doherty, Frode Lars Jahnsen, TB Trials Study Group

**Affiliations:** 1 Centre for Immune Regulation and Department of Pathology, Oslo University Hospital - Rikshospitalet and the University of Oslo, Oslo, Norway; 2 Department of Clinical Science, Faculty of Medicine and Dentistry, University of Bergen, Norway, and Department of Microbiology, Haukeland University Hospital, Bergen, Norway; 3 Aeras, Rockville, Maryland, United States of America; 4 Division of Infectious Diseases, St. John’s Research Institute, Bangalore, India; 5 Physiology and Health and Humanities, St. John’s Medical College and St. John’s Research Institute, Bangalore, India; 6 GlaxoSmithKline Pharma, Vaccines, Copenhagen, Denmark; Institut de Pharmacologie et de Biologie Structurale, France

## Abstract

**Background:**

QuantiFERON-TB Gold In-Tube (QFT) is an IFNγ-release assay used in the diagnosis of *Mycobacterium tuberculosis* (MTB) infection. The risk of TB progression increases with the magnitude of the MTB-specific IFNγ-response. QFT reversion, also associated with low Tuberculin Skin Test responses, may therefore represent a transient immune response with control of *M. tuberculosis* infection. However, studies at the single cell level have suggested that the quality (polyfunctionality) of the T-cell response is more important than the quantity of cytokines produced.

**Objective:**

To explore the quality and/or magnitude of mycobacteria-specific T-cell responses associated with QFT reversion and persistent QFT-positivity.

**Methods:**

Multi-color flowcytometry on prospectively collected peripheral blood mononuclear cells was applied to assess mycobacteria-specific T-cell responses in 42 QFT positive Indian adolescents of whom 21 became QFT negative (reverters) within one year. Ten QFT consistent negatives were also included as controls.

**Results:**

There was no difference in the qualitative PPD-specific CD4+ T-cell response between QFT consistent positives and reverters. However, compared with QFT consistent positives, reverters displayed lower absolute frequencies of polyfunctional (IFNγ+IL2+TNFα+) CD4+ T-cells at baseline, which were further reduced to the point where they were not different to QFT negative controls one year later. Moreover, absolute frequencies of these cells correlated well with the magnitude of the QFT-response.

**Conclusion:**

Whereas specific polyfunctional CD4+ T-cells have been suggested to protect against TB progression, our data do not support that higher relative or absolute frequencies of PPD-specific polyfunctional CD4+ T-cells in peripheral blood can explain the reduced risk of TB progression observed in QFT reverters. On the contrary, absolute frequencies of these cells correlated with the QFT-response, suggesting that this readout reflects antigenic load.

## Introduction

One third of the world’s population is estimated to be infected with *Mycobacterium tuberculosis* (MTB) [1]. These individuals constitute a huge reservoir for continued transmission as well as morbidity and mortality due to tuberculosis (TB). Latently infected individuals (LTBI) who do not progress to tuberculosis have been assumed to possess natural protective immunity. Therefore, the immune responses in subjects with latent MTB infection have been extensively studied in order to identify surrogate markers of protection against TB for use in vaccine efficacy trials [2], [3] and risk assessment for TB progression in LTBI subjects, enabling better targeted preventive treatment [4].

QuantiFERON-TB Gold In-Tube (QFT) is an *ex vivo* interferon gamma-release assay (IGRA) increasingly used for the diagnosis of latent MTB infection in adults, as it provides specificity superior to the Tuberculin Skin Test (TST) in populations where the BCG-vaccine is administered after infancy [4]. There is extensive evidence for a positive correlation between the size of the TST induration and TB risk [5], [6], but studies are emerging claiming equal or superior predictive power for TB outcome by IGRAs (reviewed in [4]). The risk of TB progression seems to increase with the magnitude of MTB-specific IFNγ-responses[7]–[11]. Accordingly, specific IFNγ-responses seem to be higher in subjects with active TB, where MTB is thought to be actively replicating [12], [13].

In longitudinal studies of TB exposed individuals, 20–60% of those that are IGRA-positive at baseline revert to IGRA-negative (reviewed in [4] ). IGRA reversion is more likely in subjects with a negative TST[14]–[18] and/or IFNγ-responses close to the test cut-off [14], [17]. The immunological and clinical significance of IGRA reversion is debated: Is it simply a matter of fluctuation around the test cut-off? Does MTB-reactive IFNγ-production reduce over time due to pathogen clearance or dormancy/latency? Does latent MTB occasionally secrete antigens, keeping the immune response boosted? And importantly, are reverters protected against reactivation or re-infection? (Reviewed in [19]).

Based on analogy to findings in the field of virology [20] and *Leishmania major* infection in mice [21], it has been suggested that polyfunctional CD4+ T-cells (producing IFNγ, TNFα and IL2) protect MTB infected subjects from TB progression (reviewed in [22]). The occurrence of mycobacteria-specific polyfunctional CD4+ T-cells has therefore been used to evaluate new vaccine candidates in mice [23], [24] and humans [25]. In a recent cross-sectional study it was shown that LTBI subjects had an increased proportion of mycobacteria-specific polyfunctional CD4+ T-cells in peripheral blood compared to TB patients, whereas the latter displayed a high proportion of TNFα-single-producing T-cells [26]. Although the risk of TB progression increases with the magnitude of the MTB-specific IFNγ-response [7]–[10], the findings by Harari et al (26) may suggest that the quality of the T-cell response is more important than the quantity of cytokines produced.

Supported by epidemiological studies which report reduced QFT and TST responses in subjects who later revert to QFT negative [14]–[18], we assumed that QFT reversion represents a successful immune response and reduced risk of TB progression after MTB infection. Accordingly, we hypothesized that QFT reverters differ from QFT consistent positives with regard to the quality and magnitude of MTB-specific T-cell responses in peripheral blood. By characterizing the likely favourable immune response of QFT reverters, we aimed to make a contribution in the search for biomarkers predicting TB outcome in LTBI subjects. Therefore, in the context of a prospective cohort study in Indian adolescents we compared mycobacteria-specific T-cell responses in blood samples obtained from QFT consistent positives and QFT reverters.

## Methods

### Study setting

Data and samples for the present study were collected in the context of the Adolescent Cohort Study (ACS), a prospective, observational study conducted by the TB Trials Study Group in India to establish the incidence of TB in adolescents and prepare the field site for future vaccine trials. In India, BCG vaccination is advocated at birth. The ACS enrolled 6644 adolescents aged 12–18 years from randomly selected school clusters within a typical rural/semi-urban South-Indian population (Palamaner Taluk, Andhra Pradesh). Written informed consent from parents and written assent from participants was obtained at the time of recruitment. Active TB was excluded at inclusion. The participants were randomized (by school clusters) to a 2-year active or passive surveillance. Participants with active surveillance (n = 3102) were eligible for the present study. These subjects had a TST administered and read after 48–72 hours by a trained nurse or physician (2 TU/0.1 mL tuberculin; Span Diagnostics Ltd, India) at study inclusion, day 360 and 720. QuantiFERON- TB Gold In-Tube (QFT) assay (Cellestis) was added to the original study protocol at study day 360 and 720. The QFT assay was performed on 3 ml of peripheral blood according to the manufacturer’s instructions. The subjects were clinically assessed for TB every 3^rd^ month and peripheral blood was obtained every 6^th^ month for isolation of peripheral blood mononuclear cells (PBMCs).

For the present study, healthy adolescents were randomly selected based on the QFT results from day 360 (hereafter referred to as the QFT baseline) and 1 year later (day 720). A positive QFT was defined as an IFNγ-response ≥0.35 IU/ml. 21 QFT consistent positives, 21 QFT reverters (QFT positive at baseline and QFT negative (<0.35 IU/ml) 1 year later) and 10 consistent negative controls were included. The mean age of the study participants was 13.3 years (SD 1.01) and 60% were boys. There were no difference in age or sex-distribution between QFT consistent positives, QFT reverters and QFT negative controls. Among the study subjects 46 out of 52 were BCG vaccinated (1 QFT consistent positive with missing data) whereas 36/52 had a scar present. Comparing QFT consistent positives with reverters, significantly fewer were vaccinated (16/20 versus 21/21, p = 0.048), whereas there was no difference in the presence of a BCG scar.

### Ethics Statement

The study was approved by the institutional review board at St. John’s Medical College, Bangalore, India, an independent contracted ethics review committee of the Aeras Global TB Vaccine Foundation, and the Ministry of Health Screening Committee, Government of India (No. 5/8/9/52/2006-ECD-I dt. 10.11.2006).

### PBMC sample processing

PBMCs were isolated from 16 ml peripheral blood collected on 2 Cell Preparation Tubes per subject, according to the manufacturer’s instructions (BD Biosciences). Isolated cells were resuspended in FBS (80%) with DMSO (20%) (Sigma) (volume of diluent equal to the volume of the cell suspension) and immediately frozen at −80°C for 12 hours, transferred to −152°C for typically a week, then transported to the Biorepository liquid N_2_ freezers at St. John’s Research Institute. Selected samples were then shipped in liquid N_2_ (MVE Vapor Shipper) to Oslo University Hospital. PBMC were thawed by drop-wise resuspension in preheated RPMI 1640 (BioWittakre) with 10% FBS (Sigma) and 1% L-glutamine (Lonza, BioWhittaker) and left over-night. Cell counts and viability were assessed on Tryphan Blue (Invitrogen) stained cells (Countess, Invitrogen) and in some samples confirmed by manual counts (Acridine Orange, Sigma). Only samples with cell viability >70% were used for further analysis.

### In vitro stimulation

PBMCs were assessed for *ex vivo* responses to peptide pools of Early Secretory Antigen Target-6 (ESAT-6) (2 µg/mL) and Purified Protein Derivate (PPD) (10 µg/mL), both provided by Statens Serum Institute, Copenhagen, Denmark. Staphococcal Enterotoxin B (SEB; Sigma) and PBS were used for positive and negative controls, respectively. Approximately 1 million PBMCs in a total volume of 300 µL were stimulated for 6 hours in 96-well round bottom plates (Corning Life Sciences) in the presence of αCD28 (10 µg/mL, clone L293, BD) and αCD49d (10 µg/mL, clone L25, BD). Samples from the same subject were set up side by side in the same plate. Brefeldin A (1.0 µl/well) and monensin (0.7 µl/well) (both from BD) were added to the cell-antigen mix prior to the 6 h incubation.

### Multi-color staining protocol

As CD107a is a degranulation marker, αCD107a-FITC (BD), was added together with the Golgi inhibitors (BD) prior to incubation. After stimulation, samples were washed in PBS, stained with LIVE/DEAD Violet Fixable Dead Cell Stain (Invitrogen) for 10 min, washed with PBS, and then stained with αCD3-APC Alexa Fluor 750 (Beckman Coulter), αCD4-PerCP Cy5.5 (BD), αCD14-PacBlue (BD), CD16-PacBlue (BD), αCD19-PacBlue (Invitrogen) and αCD45RO-PE Cy5 (BD) for 25 min, washed twice with FACS buffer (PBS with 2% FBS and 0.01% sodium azide), fixed and permeabilized for 10 min using Cytofix/Cytoperm and Perm/Wash Buffer (BD). The cells were then stained with IFNγ-Alexa Fluor 700 (Invitrogen), IL2-APC (BD), TNFα-PE Cy7 (BD), and perforin-PE (Diaclone) for 30 min and washed twice with Perm/Wash Buffer. Stained samples were stored in 1% formaldehyde at 4°C until flowcytometric analysis (performed within 48 hours). All staining steps and incubations were performed at room temperature.

### Flowcytometric analysis

All samples were run on a BD LSR II flow cytometer (BD Biosciences), and analyzed using FlowJo Software (version 9.4.1, Three Star Inc). BD CompBeads were used for compensation. For each sample, 20 000 events were recorded for quality assessment, before the storage gate was set to a wide lymphocyte gate based on forward- and side scatter properties. (Gating strategy is provided as Figure S1). Samples were excluded from the study if the lymphocyte gate was poorly defined; if the CD3+CD4+ or CD3+CD4- cell counts were less than 5000; or if the sample was lacking a valid positive control (>1% cytokine-producing cells when stimulated with SEB). When assessing cytokine–positive cells, the background response to PBS was subtracted. A threshold for a positive cytokine response was set at a 2-fold increase in cell count above the background, and samples that did not meet this requirement were set to zero.

### Statistical analysis

The distribution of age, sex and BCG-status between the QFT categories was assessed by ANOVA and Fisher’s Exact test where appropriate. For TST, QFT and absolute frequencies of T-cell subsets (flowcytometric data), non-parametric statistics were applied as the data were not normally distributed. The TST and QFT data were assessed by Mann-Whitney U test and Wilcoxon Signed rank test for unpaired and paired testing, respectively. As the flowcytometric data on relative frequencies are normalized, parametric statistics (unpaired T-test with Welch’s correction) were applied [27]. Correlations were assessed by Spearman’s Rank Order Correlation. A two-tailed α-level of p<0.05 was considered significant. Microsoft Office Excel 2003 and GraphPad Prism version 5 were used for data handling and statistical analyses.

## Results

### QuantiFERON-TB Gold In-Tube and Tuberculin Skin Test responses in QFT consistent positives, reverters and negative controls

**Figure 1:**
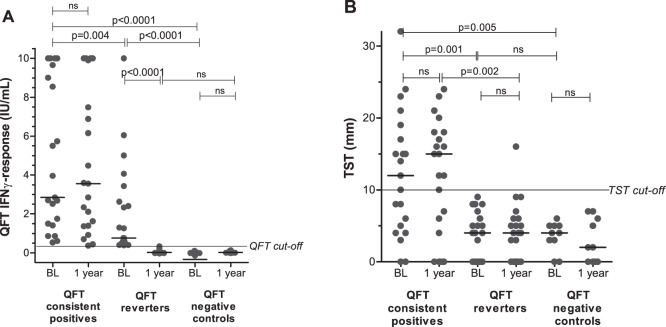
QuantiFERON Gold In-Tube (QFT) and Tuberculin Skin Test (TST) responses. (**A**) The magnitude of the QFT response and (**B**) the TST response in QFT consistent positives (n = 21), QFT reverters (n = 21) and QFT negative controls (n = 10) at baseline (BL) and after 1 year. Unpaired and paired analyses was performed with Mann-Whitney U test and Wilcoxon Signed rank test where appropriate. Horizontal lines represent median values.

At baseline, the magnitude of QFT and TST responses were higher in QFT consistent positives compared to QFT reverters (Figure 1A and 1B) and both were significantly higher than responses in the QFT consistent negative controls. Among consistent positives, 19 of 21 had QFT values ≥0.70 IU/ml, a suggested upper limit for a “grey-zone” (0.35 to 0.70 IU/mL) in test interpretation [14], whereas 18 of 21 reverters had either a baseline value ≥0.70 IU/ml or a reduction in IFNγ-release of ≥80% from baseline to the reversion one year later, suggestion true test reversion [28]. At one year after the first test, the magnitude of the response in the reverter group was indistinguishable from the QFT-negatives. More than half of the consistent positives showed a positive TST at both time points, whereas all reverters had a TST below the cutoff level of 10 mm at baseline. After one year, one reverter had a TST ≥10 mm (Figure 1B).

### Characterization of polyfunctional mycobacteria-specific CD4+ T-cell responses in QFT consistent positives and reverters

We tested all samples for viability and only samples with a cell viability of >70% were used for the subsequent analyses. Available PBMC samples were investigated with regard to cytokine responses in CD4+ T-cells as well as cytokine responses and cytotoxic capacity in CD8+ T-cells. As previously reported, the number of CD8+ T-cells responding to PPD or ESAT-6 were few [29], and the responses weak [30], [31] (not shown). Consistent with others [32], we also observed few responding cells among CD45RO^low^ CD4+ T-cells (not shown), which are mainly naïve T-cells. In the following we therefore focused on the CD4+CD45RO+ T-cell subset, which are mainly memory T-cells.

**Figure 2:**
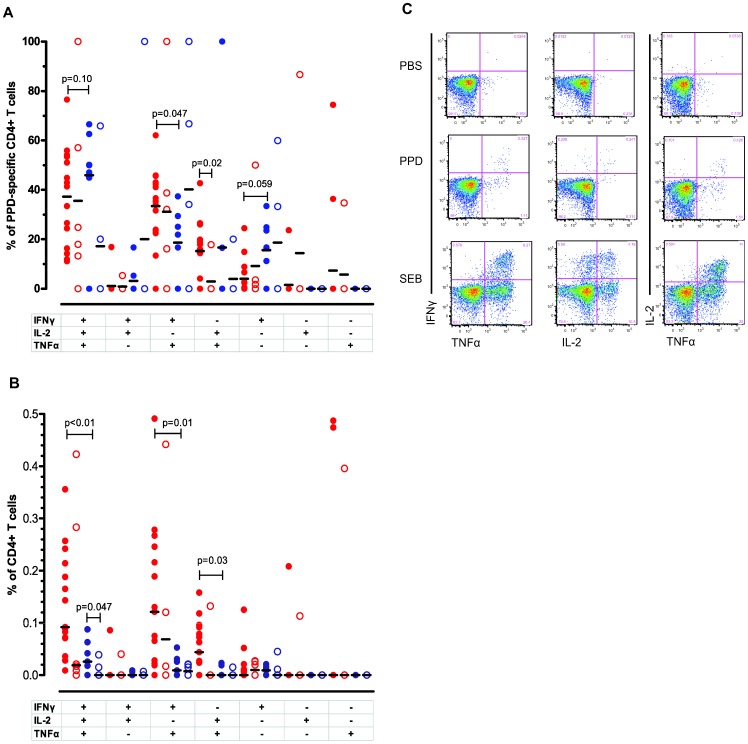
Qualitative and quantitative analysis of PPD-specific CD4+CD45RO+ T-cell responses in QFT consistent positives and QFT reverters. (**A**) The relative frequencies of the PPD-specific CD4+CD45RO+ T-cell subsets in QFT consistent positives (red symbols) and QFT reverters (blue symbols) at baseline (filled) (n = 16, n = 7) and after 1 year (open) (n = 6, n = 6). The subsets are defined on the basis of IFNγ, TNFα and/or IL-2 production. Since relative frequencies are normalized data, un-paired T-test with Welch’s correction was applied for statistical analyses and horizontal lines represent mean values. (**B**) The absolute frequencies of PPD-specific CD4+CD45RO+ T- in QFT consistent positives (red symbols) and QFT reverters (blue symbols) at baseline (filled) (n = 16, n = 7) and after 1 year (open) (n = 6, n = 6). As the data are not normally distributed, statistical analyses were performed by the Mann-Whitney U test and horizontal lines represent median values. (**C**) Flowcytometric plots of the functional profile of PPD-specific CD4+CD45RO+ T-cells compared with negative (PBS) and positive (SEB) control in one representative QFT consistent positive subject are shown.

We first asked whether there were qualitative differences in mycobacteria-specific CD4+CD45RO+ T-cell responses between QFT consistent positives and reverters at baseline. To assess this we analyzed the relative frequencies of triple, double and single cytokine-producing CD4+ T-cells after 6 hours of stimulation with either PPD or ESAT-6. A high proportion of PPD-reactive CD4+ T-cells were polyfunctional (IFNγ+IL2+TNFα+) in both QFT consistent positives and reverters (median 36% and 59%, respectively: not significantly different). The proportion of PPD responsive IFNγ+IL2+TNFα+ memory T-cells in both groups had decreased one year later, most obviously in the reverter group, but none of these changes were significant. QFT reverters had a reduced proportion of PPD-reactive IFNγ+TNFα+ CD4+ T-cells compared to the consistent positives at baseline (Figure 2A). One year later, the proportion of IL2+TNFα+ CD4+ T-cells in QFT consistent positives fell compared to baseline (reduction in median from 16% to 0%, p = 0.02). Other subsets did not show any significant differences between the groups (Figure 2A).

The quantitative differences were more profound. Although (as noted above) the relative percentages of polyfunctional CD4+ T-cells were very similar, the magnitude of the response was greater in the QFT consistent positive group, which at baseline had significantly higher numbers (absolute frequencies) of PPD-responsive IFNγ+IL2+TNFα+ CD4+ T-cells, IFNγ+TNFα+ CD4+ T-cells and IL2+TNFα+ CD4+ T-cells. Although the same trend of decreasing responses after 1 year was visible in the quantitative assessment, this was only significant for IFNγ+IL2+TNFα+ CD4+ T-cells from the QFT reverter group (Figure 2B).

With regard to ESAT-6-reactive CD4+ T-cells, we observed similar trends for relative and absolute frequencies as seen for PPD, but as some samples did not respond to ESAT-6 and, consistent with previous studies [29], [30], [33], the responses were generally weaker and significance was not obtained for these changes (data not shown).

### Quantitation of IFNγ, IL2 and TNFα production on a per cell basis in PPD-specific CD4+ T-cells

**Figure 3:**
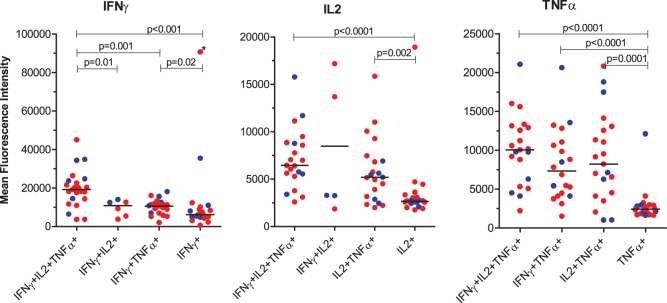
The magnitude of cytokine production by single, double and triple-producing MTB-specific CD4+CD45RO+ T cells at baseline. The magnitude of IFNγ, IL2 and TNFα expression measured by mean fluorescence intensity (MFI), by single, double and triple-producing CD4+CD45RO+ T cell subsets in response to PPD is shown. QFT consistent positives and reverters are indicated by red or blue color, respectively. Horizontal lines represent median values. Statistical analysis was performed by Mann-Whitney U test.

We then asked whether the amount of cytokine produced on a single cell level was related to polyfunctionality. To assess this we measured the mean fluorescence intensity (MFI) of IFNγ, IL2 and TNFα by triple, double, and single cytokine-producing CD4+ T-cells after stimulation with PPD (Figure 3). In most situations, single-producing T-cells produced significantly less cytokine compared with subsets producing two or three cytokines simultaneously. Importantly, with regard to IFNγ- production, polyfunctional CD4+ T-cells produced significantly more IFNγ than all the other cytokine-producing cell subsets (Figure 3). However, there were no significant differences in MFI between consistent positives and reverters.

### The absolute frequency of PPD-specific IFNγ-producing CD4+ T-cells correlated well with the QuantiFERON-TB Gold In-Tube response

**Figure 4:**
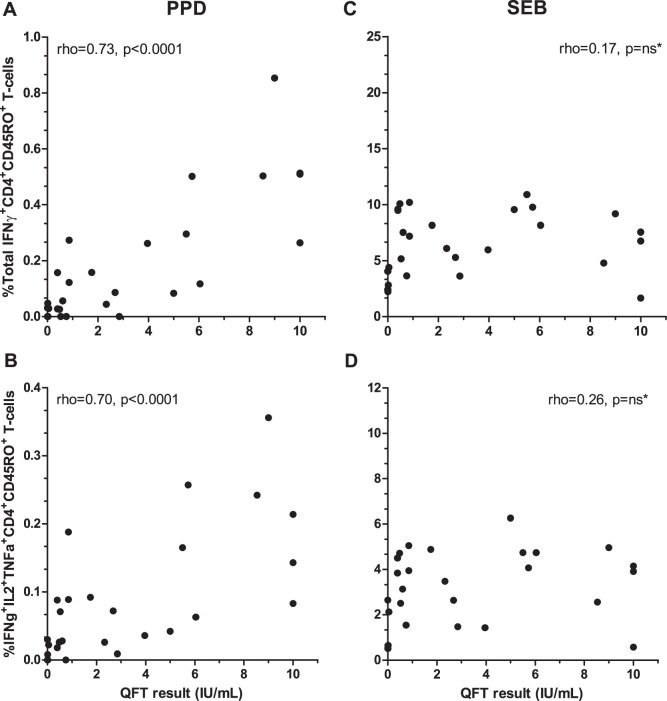
The relationship between the cytokine-producing CD4+CD45RO+ T cells and the QuantiFERON Gold In-Tube (QFT) response at baseline. The relationship between the absolute frequencies of (**A**) all IFNγ-producing and (**B**) polyfunctional (IFNγ, IL2 and TNFα) PPD-specific CD4+CD45RO+ T cells and the magnitude of the QFT response are shown. The relationship between the absolute frequencies of cytokine-producing T-cells in response to SEB is shown for comparison (**C and D**). Statistical analyses were performed by Spearman’s correlation coefficient (rho).

As the QFT test measures the total amount of secreted IFNγ in response to MTB-specific antigens, we then asked whether the magnitude of the QFT response correlated with the production of IFNγ at the single cell level, when applying PPD, a less specific antigen. Should this be the case, the differences in PPD-specific polyfunctional CD4+ T-cells described between QFT consistent positives and reverters would very likely be true, despite the limited sample size and spread in the data for QFT consistent positives after 1 year. As shown in Figure 4, both the absolute frequency of all PPD-specific IFNγ-producing T-cells as well as the absolute frequency of polyfunctional T-cells correlated well with the QFT-response. Notably, these correlations were likely to be underestimated as serial dilutions for QFT responses >10 IU/mL were not performed. In comparison, no correlation could be found for SEB stimulated cells and the QFT response (Figure 4C–D). This indicates: Firstly, that the polyfunctional T-cell responses to PPD measured here, reflects specific anti-MTB immune reactivity. Secondly, that the absolute frequency of PPD-specific polyfunctional CD4+ T-cells are likely to vary in concordance with the QFT response, hereby supporting a decline in conjunction with QFT reversion.

## Discussion

To our knowledge, this is the first prospective study designed to assess the dynamics of mycobacteria-specific T-cell responses at the single cell level in QFT consistent positives compared to QFT reverters. Our results showed that the proportion (relative frequency) of PPD-specific polyfunctional (IFNγ+IL2+TNF+) CD4+ T-cells was high in both groups. Except for a small difference in the proportion of IFNγ+TNFα+ CD4+ T-cells (QFT consistent positives>reverters), we found no differences in the relative frequency of cytokine-producing CD4+ T-cells at baseline between the groups. With regard to absolute frequencies, QFT reverters displayed lower numbers of polyfunctional (IFNγ+IL2+TNF+) and double-producing (IFNγ+TNFα+ and IL2+TNFα+) CD4 T-cells. One year later the PPD-specific T-cell responses remained unchanged in QFT consistent positives, whereas QFT reverters had reduced absolute frequencies of polyfunctional T-cells which were not different from QFT negative controls.

It has been suggested that polyfunctional CD4+ T-cells are important mediators of protective immunity in MTB infected subjects. Moreover, QFT reversion has been suggested as a good prognostic marker with regard to risk of TB progression in LTBI subjects[14]–[18]. We therefore hypothesized that the quality and/or the magnitude of mycobacteria-specific polyfunctional T-cells were higher in QFT positive subjects who later became QFT negative compared to consistent positives. We found that both QFT consistent positives and reverters had high relative frequencies of polyfunctional CD4+ T-cells, but observed no difference between the groups at any time point. Importantly, the distribution of the PPD-specific cytokine-producing T-cells in both QFT consistent positive and reverters reported here is comparable with findings in the LTBI group reported by Harari et al., and very different from TB patients in the same study [26]. However, our findings do not support the hypothesis that increased relative frequencies of polyfunctional mycobacteria-specific T-cells is associated with the reduced risk of TB progression seen in QFT reverters [14]–[18].

To our knowledge, no prospective studies on LTBI adults have evaluated the protective effect/prognostic value of polyfunctional mycobacteria-specific T-cells, but results from cross-sectional studies comparing LTBI subjects with TB patients are used as arguments for or against a protective effect of polyfunctional CD4+ T-cells in TB progression [22], [30]–[32], [34]. The only prospective study in humans so far published was performed on BCG vaccinated infants and concluded that the absolute frequencies of mycobacteria-specific polyfunctional CD4+, CD8+ and γδ+ T-cells did not correlate with TB protection [35]. Interestingly, in a mouse model of vaccine-induced immunity, the absolute frequencies of polyfunctional lung-resident mycobacteria-specific CD8+ and CD4+ T-cells correlated with protection whereas high levels in the spleen did not [24], indicating that tissue-specific distribution might be important. We found that QFT reverters had lower absolute frequencies of PPD-specific polyfunctional CD4+ T-cells in peripheral blood at baseline compared to consistent positives. Furthermore, the frequencies of the polyfunctional T-cells in reverters were further reduced after one year (and became not significantly different from QFT negative controls). As opposed to the continued presence of mycobacteria-specific polyfunctional CD4+ T-cells in QFT consistent positives, our findings in the reverter group likely reflects a transient immune response after MTB exposure.

We also found that PPD-stimulated CD4+ T-cells expressing more than one cytokine produced more cytokine on a per cell basis than single-positive cells. This is in keeping with the concept that polyfunctional T-cells have a stronger protective effect in MTB-infection [22]. Therefore, it might be surprising that few studies support the concept that high proportions and/or high absolute frequencies of these cells in peripheral blood in latency are correlates of protection from TB progression. This may be because the presence and localization of polyfunctional T-cells at infection seem crucial as high numbers in the lung shortly after infection are protective [2], [24], [36], [37] by their capacity to restrict bacterial replication and thus promote clearance or successful containment [38]–[40]. In this regard, one might argue, that in case of re-infection, QFT consistent positives are more likely than reverters to have adequate numbers of circulating memory T-cells capable of rapid elimination/containment of new MTB bacilli. On the other hand, mouse models suggest that repeated mycobacterial exposure (BCG-vaccination) in already MTB infected mice increased the lung damage independent of the expression of IFNγ in the lesion[41]–[43].

The QFT assay measures the total production of IFNγ in response to three MTB-specific antigens and does not differentiate between different types of cytokine-producing T-cells. As we report good correlation between the magnitude of the QFT response and the absolute frequencies of PPD-specific polyfunctional CD4+ T-cells, the rather simple QFT assay, seems, in fact, a good surrogate marker for the numbers of mycobacteria-specific (probably MTB-specific) polyfunctional T-cells in peripheral blood of LTBI individuals. This finding questions the rationale for performing elaborate flow cytometry assays on peripheral blood samples in the search for “protective biomarkers”. The finding that QFT levels correlated well with the total number of PPD-specific IFNγ-producing T-cells further strengthens the validity of our results, and suggests little interference by BCG-vaccination and NTM-exposure. Notably, BCG-vaccination at birth has little influence on the TST response [4].

## Conclusions

Our findings show that mycobacteria-specific polyfunctional (IFNγ+IL2+TNF+) CD4+ memory T-cells are the most frequent cytokine-producing cells in LTBI individuals and that these cells are very efficient cytokine-producers compared to single-cytokine producing T-cells. Contrary to our starting hypothesis, our data do not support that the relative or absolute frequencies of PPD-specific polyfunctional CD4+ T-cells in peripheral blood can explain the reduced risk of TB progression observed in QFT reverters. Contrary, absolute frequencies of these cells correlated with the QFT-response, suggesting that this readout reflects the antigen load, similar to that shown for IGRA-responses in non-human primates [44]. Our data also supports earlier speculations on the requirement for persistent or recurrent MTB exposure to maintain specific IFNγ-responses [15], [16], [18], [45]. The mechanisms behind QFT reversion requires further exploration. Notably, we cannot draw firm conclusions on the risk of TB progression in QFT consistent positives. Both QFT consistent positives and reverters were infected at least one year before the end time point of this study, but progression to TB at a later time point cannot be excluded. The TB Trials Study group has prospectively collected longitudinal PBMC samples from the 6644 subjects enrolled in the ACS, amongst whom some progressed to TB during the 2-year surveillance. Work on this analysis is ongoing and hopefully, these samples will allow us to identify biomarkers to predict TB progression in LTBI subjects.

## Supporting Information

Figure S1 **Gating Strategy.**
(EPS)
